# Progress towards a representative network of Southern Ocean protected areas

**DOI:** 10.1371/journal.pone.0231361

**Published:** 2020-04-22

**Authors:** Cassandra M. Brooks, Steven L. Chown, Lucinda L. Douglass, Ben P. Raymond, Justine D. Shaw, Zephyr T. Sylvester, Christa L. Torrens

**Affiliations:** 1 Environmental Studies Program, University of Colorado, Boulder, Boulder, CO, United States of America; 2 School of Biological Sciences, Monash University, Melbourne, Australia; 3 Centre for Conservation Geography, Sydney, New South Wales, Australia; 4 Centre for Biodiversity and Conservation Science, School of Biological Sciences, The University of Queensland, Brisbane, Queensland, Australia; 5 Australian Antarctic Division, Department of the Environment, Kingston, Tasmania, Australia; Centre National de la Recherche Scientifique, FRANCE

## Abstract

Global threats to ocean biodiversity have generated a worldwide movement to take actions to improve conservation and management. Several international initiatives have recommended the adoption of marine protected areas (MPAs) in national and international waters. National governments and the Commission for the Conservation of Antarctic Marine Living Resources have successfully adopted multiple MPAs in the Southern Ocean despite the challenging nature of establishing MPAs in international waters. But are these MPAs representative of Southern Ocean biodiversity? Here we answer this question for both existing and proposed Antarctic MPAs, using benthic and pelagic regionalizations as a proxy for biodiversity. Currently about 11.98% of the Southern Ocean is protected in MPAs, with 4.61% being encompassed by no-take areas. While this is a relatively large proportion of protection when compared to other international waters, current Antarctic MPAs are not representative of the full range of benthic and pelagic ecoregions. Implementing additional protected areas, including those currently under negotiation, would encompass almost 22% of the Southern Ocean. It would also substantially improve representation with 17 benthic and pelagic ecoregions (out of 23 and 19, respectively) achieving at least 10% representation.

## Introduction

Global threats to ocean biodiversity have generated worldwide momentum to improve its conservation and management. Marine protected areas (MPAs), areas of ocean where human activities are limited or prohibited, have been increasingly promoted by policy-makers, scientists and conservationists as a tool for mitigating ocean threats, conserving biodiversity, and managing fisheries [1–3]. Numerous studies demonstrate that MPAs, especially no-take MPAs (also known as marine reserves), lead to increases in biomass, density, and diversity of life in the MPA [4–6]. Notably, these MPA benefits can extend to fisheries. MPAs have been shown to facilitate the recovery of depleted fisheries, provide spillover effects, and lead to larger fish [7–9]. Furthermore, because they maintain all trophic levels of the ecosystem and increase both species and genetic diversity, MPAs can enhance resilience to environmental impacts, including those related to climate change [10–12].

Several international targets have recommended the adoption of representative networks of MPAs in national waters and in areas beyond national jurisdiction. At the 2002 World Summit on Sustainable Development, participating States agreed to designate a representative global network of MPAs by 2012 [13]. This call was further reiterated at the 2003 International Union for the Conservation of Nature (IUCN) World Parks Congress, which called for protected areas encompassing 20–30% of all marine habitats also by 2012 [14]. The 2010 Aichi Biodiversity Targets, adopted by the Convention on Biological Diversity as part of its Strategic Plan for Biodiversity 2011–2020, offered a new deadline of 2020 to designate 10% of the global oceans in ecologically representative MPAs [15]. Then, in 2014 the IUCN World Parks Congress recommended that 30% of the ocean be protected in an ecologically representative network [16]. Finally, in 2015 the United Nations adopted the Sustainable Development Goals, including goal 14 which aims to conserve 10% of coastal and marine areas by 2020 [17]. Evidence-based conservation science research often suggests protection targets of at least 30% and often higher are required to effectively conserve biodiversity and ecosystems [18, 19].

A major criteria for conservation, including Aichi target 11, is that protected areas be ecologically representative since their efficacy is substantially enhanced when they are representative of the biodiversity of a region [20]. A widely used method for examining representivity is by determining coverage of ecoregions by the protected area network [21, 22]. Ecoregions are spatial regions, typically within a large spatial domain, defined in such a way that each ecoregion defines a characteristic set of species communities and habitats that are distinct from those of other ecoregions within the domain [23, 24]. Direct sampling of biodiversity at large spatial scales is generally impractical, necessitating the use of proxies or modelling approaches to achieve broad spatial coverage. Species distribution and related modelling methods can be used to infer broad-scale biodiversity patterns based on spatially-limited sampling (e.g.[25, 26]). However, such approaches present difficulties for our purposes of assessing Southern Ocean MPA representativeness. Predictions of species distributions would need to be available at circum-Antarctic scale, and from a sufficiently diverse suite of species in order to be suitably representative of broader Southern Ocean biodiversity To date, Southern Ocean applications of such models have tended to be regional in scope (but see e.g. [25, 27–30] for circum-Antarctic applications), and focused on a relatively restricted number of species. Here we therefore use heterogeneity of habitats and geomorphic features as proxies for biodiversity. This approach is well established in the terrestrial and marine realms (see e.g., [31–35]).

In line with global MPA goals, roughly 18.45% of national waters, globally, have been protected to date. Meeting these targets in areas beyond national jurisdiction has proven a more difficult challenge, with only 1.18% of the high seas protected thus far [36]. Further, MPAs have generally been found to not be ecologically representative, especially within waters under national jurisdiction [37, 38].

**Fig 1:**
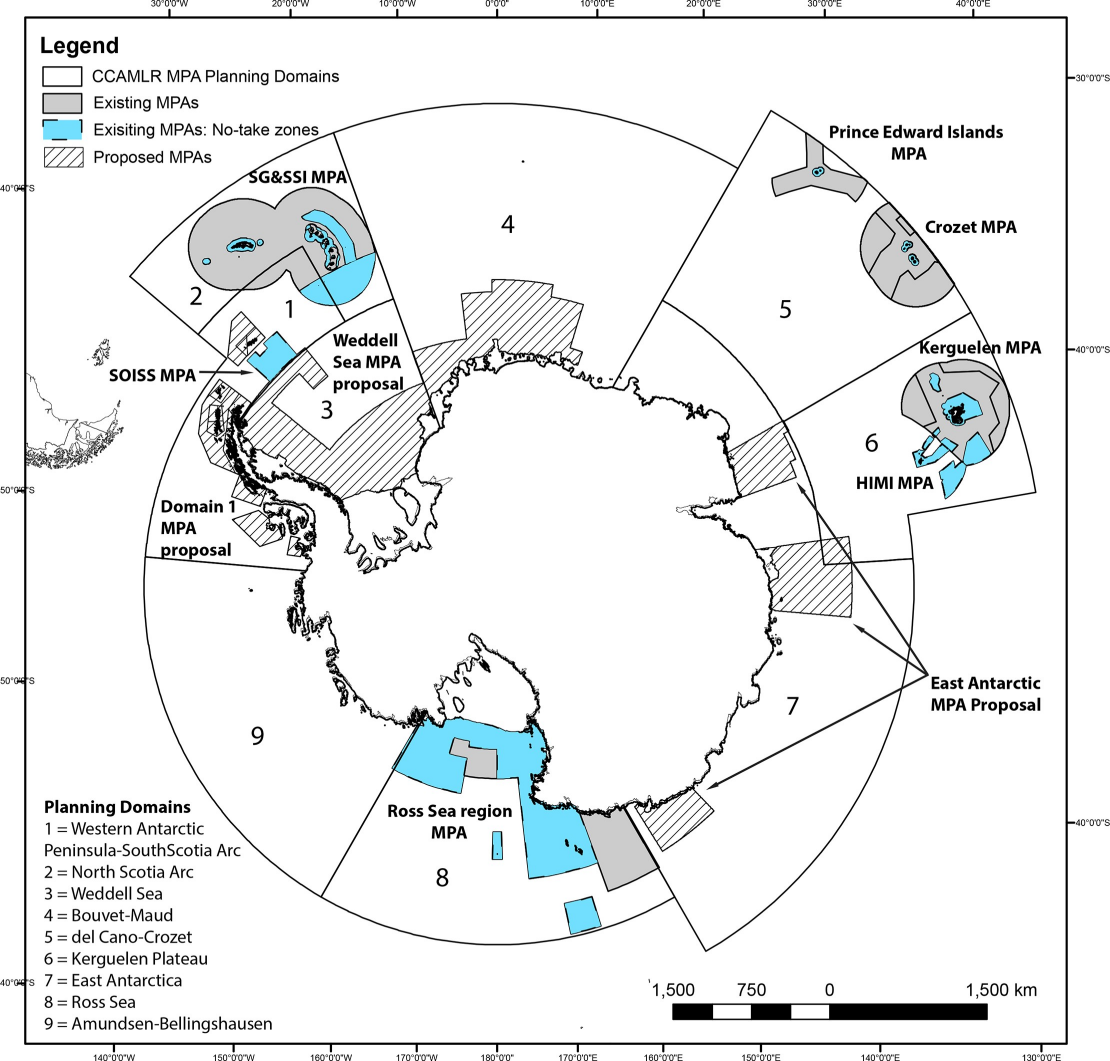
CCAMLR planning domains and marine protected areas in the Southern Ocean. CCAMLR MPA planning domains (outlined in black line) and MPAs in the Southern Ocean. Existing MPAs are coloured grey (outlined in solid line) with no-take areas in blue (outlined in dotted line) and proposed MPAs represented by hashed lines. SOISS refers to South Orkney Islands Southern Shelf, SG&SSI refers to South Georgia and South Sandwich Islands, HIMI refers to Heard Island and McDonald Islands. (CCAMLR MPA planning domains and MPA boundaries from [48]; sub-Antarctic MPA boundaries from [36]; East Antarctic proposed MPA boundaries based on [49]; Weddell Sea MPA boundaries from [50]; and Domain 1 proposed MPA boundaries [51], the latter two with permission).

National governments and the Commission for the Conservation of Antarctic Marine Living Resources (CCAMLR) have successfully adopted multiple MPAs in the Southern Ocean despite the challenging nature of establishing MPAs in international waters. The Southern Ocean encompasses roughly 10% of the global oceans (Fig 1), most of which is considered high seas. This area is primarily governed by a multi-lateral Convention on the Conservation of Antarctic Marine Living Resources (CAMLR Convention). This Convention is carried forward by CCAMLR, a Commission of 25-Member States plus the European Union. Within CCAMLR’s waters are five sets of sub-Antarctic islands that fall under national jurisdictions (Fig 1), which are managed in accordance with Convention rules [39]. CCAMLR has the explicit objective to conserve marine living resources and employs a science-based precautionary and ecosystem-based management approach [39]. In doing so CCAMLR is arguably the world’s most successful international management body for marine living resources [40–43].

In 2002, in an effort to meet global MPA targets and in recognition of the value of MPAs as a biodiversity conservation and fisheries management tool, CCAMLR committed to designating a network of MPAs in the Southern Ocean [44]. Since 2002, CCAMLR and its Member States have held a variety of MPA workshops [44]. CCAMLR scientists, independent experts and conservation organizations have also conducted extensive Southern Ocean regionalizations, identifying areas of potential importance for biodiversity and ecosystems [24, 45, 46]. These benthic and pelagic regionalizations have helped guide CCAMLR towards identifying priority areas to be incorporated into a representative network of Southern Ocean MPAs [47].

Over the last two decades, CCAMLR and states with jurisdiction over sub-Antarctic islands have been designating MPAs (Fig 1; Table 1). First in 2002, Australia declared an MPA around the sub-Antarctic Heard Island and McDonald Islands, which was expanded in 2014 (~71,000 km^2^) [52]. In 2006, France designated MPAs around the sub-Antarctic Crozet and Kerguelen Islands which were extended in 2017 (combined ~ 580,000 km^2^) [53]. In 2009, CCAMLR adopted its first high sea MPA south of the South Orkney Islands (~94,000 km^2^) [54]. In 2012, the United Kingdom declared a large MPA around the South Georgia and South Sandwich Islands, which was expanded in 2019 (~1.24 million km^2^) [55]. Furthermore in 2013, South Africa designated an MPA around the sub-Antarctic Prince Edward Islands (161,000 km^2^) [56, 57]. In 2016, CCAMLR adopted the world’s largest international MPA in the Ross Sea (~1.5 million km^2^) [58]. Additionally, CCAMLR has been negotiating a large MPA network in the East Antarctic, the Weddell Sea, and the Antarctic Peninsula (Fig 1). Adjacent to the CCAMLR Area other sub-Antarctic MPAs exist (e.g., Macquarie Island Marine Park,162,000 km^2^ declared in 1999) however these are outside of CCAMLR jurisdiction.

**Table 1:** Established and proposed MPAs in the CCAMLR Area, including sizes, no-take areas, and benthic ecoregions and pelagic clusters encompassed by the MPAs (all constrained to the CCAMLR Area). SG&SSI refers to South Georgia and South Sandwich Islands, HIMI refers to Heard Island and McDonald Islands.

EXISTING MPAS	area (km^2^)	no-take area (km^2^)	Benthic ecoregions	Pelagic clusters
Ross Sea region	1,525,651*	1,092,788	A, O, PAR, PB, RS	1, 2, 3, 4, 5, 6, 7, 8, 9, 10, 11, 12, 14, 15, 16
South Orkney Islands	93,751	93,751	AB, SOI	1, 3, 7, 9, 10, 11, 12
HIMI	70,560	70,560	K-DK, K-KP	13, 14, 15, 16
Prince Edward Islands	160,784	4,433	AB, DC	13, 15, 16, 17, 19, 20
Kerguelen	567,978	110,650	K-DK, K-KP	13, 14, 15, 16, 17, 20
Crozet	410,450	9,158	DC, OL	13, 16, 17, 20
SG & SSI	1,241,295	284,197	AB, SG, SOI, SSI	1, 2, 3, 7, 9, 10, 11, 12, 13, 14, 15, 16
**TOTAL**	4,070,468	1,665,537		
**% CCAMLR Area**	11.98	4.90		
**PROPOSED MPAs**				
East Antarctic	969,000		AB, CI-EK, CI-PB, CI-W, CI-WK, EIA, K-BB, K-DK, O	1, 2, 3, 4, 5, 6, 7, 8, 9, 10, 11, 12, 15
Weddell Sea	1,968,175		AB, AP, DM, SOI, WS	1, 2, 3, 4, 5, 6, 7, 8, 9, 10, 11, 12
Domain 1	466,000		A, AP, SOI	1, 2, 3, 4, 5, 6, 7, 8, 10, 11, 12, 13, 14, 15
**TOTAL** (Existing + Proposed)	7,473,643			
**% CCAMLR Area**	21.99			

*The Ross Sea region MPA includes the area under the Ross Ice Shelf, however, due to lack of data under the Ice Shelf, we did not include this area in our analysis. The size of the Ross Sea MPA when including the area under ice shelves is greater than 2 million km^2^.

Here we assess progress towards establishing a representative network of MPAs, including its level of protection, in the Southern Ocean. We examine CCAMLR and nationally governed protected areas that have been adopted as well as those currently under negotiation. We map the location of existing and proposed MPAs and calculated no-take areas. We then assess whether these proposed and existing MPAs are representative of Southern Ocean biodiversity and ecosystems using existing benthic and pelagic regionalizations as a proxy for biodiversity [45, 46].

## Materials and methods

Here, we use the CAMLR Convention Area as our study region (Fig 1). This region is circumpolar, with its northern boundary between 60 and 45°S aligning approximately with the Polar Front and its southern boundary aligning with the coast of Antarctica and ice shelf boundaries. Due to the lack of data under ice shelves, our study area does not include areas under ice shelves (thus it omits the area under the Ross Ice Shelf, which is technically part of the MPA) nor does it include some of the sub-Antarctic region situated above the Polar Front which falls outside the bounds of the CCAMLR Convention Area.

### Benthic regionalization

Benthic ecoregion data representing 23 different categories [45] were downloaded from [59]. The benthic regionalization was based on a previously published hierarchical classification [31] and included ecoregions, bathomes and environmental types [45]. However, only the broadest scale unit–ecoregions–were included in this analysis. These previously published ecoregions were established based on patterns of endemism, recent biogeographic research and consideration of the influence of environmental drivers as potential barriers to dispersal [45]. A variety of previously published circumpolar datasets were used within the benthic classification, including depth [60], geomorphology [61], seafloor temperature [62], sea-surface chlorophyll [63], sea ice concentration [64] and frontal systems [65]. Previously defined regions and boundaries were also incorporated, including those regarding the Antarctic continental shelf and slope [24, 66–69], as well as patterns of endemism [66, 68] (Table 2). The data were bound by the CCAMLR Convention area. For further details on methods underpinning the generation of the benthic ecoregion data used in this analysis see [45].

**Table 2:** Benthic ecoregions of the Southern Ocean, including abbreviations, descriptions (from [45]) and percentages of the benthic ecoregions included in: No-take zones of existing MPAs; existing MPAs; and existing and proposed MPAs combined.

Benthic ecoregion	Abbrev-iation	Description	% in no-take	% in existing MPAs	% in MPAs + proposals	Size of ecoregion (km^2^)
Amundsen	A	Productive shelf & polynyas of Amundsen & Bellingshausen seas. Oceanic shallow environments of Peter I Island, De Gerlache Seamounts & Marie Byrd Seamount group.	0.21	0.21	1.15	1,550,758
Antarctic Peninsula	AP	Shallow, productive shelf of west Antarctic Peninsula with a low duration of sea ice cover & warm seabeds relative to other Antarctic shelf areas. Island ecosystems of South Shetland Islands. 13 endemic molluscs. >10% of gastropods endemic.	0	0	46.46	910,737
Atlantic Basin	AB	Very deep & very cold rugose ocean floor & abyssal plain of South Atlantic Ocean Basin & Weddell Sea.	1.54	2.36	11.26	7,134,098
Central Indian—East Kerguelen Subregion	CI-EK	Central Indian region of East Antarctica influenced by Kerguelen Plateau including downstream productivity from frontal activity across Plateau.	0	0	50.67	558,681
Central Indian—Prydz Bay Subregion	CI-PB	Central Indian region of East Antarctica that contains the cold, productive waters of Prydz Bay & Prydz Gyre which oceanographically separates east & west Kerguelen Central Indian subregions.	0	0	16.41	455,342
Central Indian–West Kerguelen Subregion	CI-WK	Central Indian region of East Antarctica not influenced by Kerguelen Plateau nor Weddell Gyre.	0	0	68.43	173,556
Central Indian—Wilkes Subregion	CI-W	Central Indian region of East Antarctica oceanographically separated from East Kerguelen subregion.	0	0	4.42	486,762
Del Cano	DC	Shallow, warm seabeds in sub-Antarctic Frontal Zone including South West Indian Ridge seamounts, Del Cano Rise & Crozet & Prince Edward Islands.	1.50	56.91	56.91	908,603
Dronning Maud	DM	Maud Rise & associated open ocean polynya, Astrid Ridge, Gunnerus Ridge & canyons offshore Dronning Maud Land. Easternmost extent of Weddell Gyre. 20 endemic molluscs (19% of documented species). 21% of documented gastropods are endemic.	0	0	34.75	673,365
East Indian Abyssal	EIA	The very deep and cold seabeds of rugose ocean floor & abyssal plains of South Indian Ocean Basin.	0	0	3.59	2,880,769
Kerguelen—Banzare Bank Subregion	K-BB	Shallower (mostly depths between 1000–3000 m), warmer seabeds of Banzare Bank, south of frontal activity of Fawn Trough.	0	0	12.46	270,266
Kerguelen—Deep Kerguelen Subregion	K-DK	Deep (mostly depths >3000 m) ocean surrounding Kerguelen Plateau & Banzare Bank.	3.57	13.39	15.97	1,807,252
Kerguelen—Kerguelen Plateau Subregion	K-KP	Shallower (mostly depths between 200–3000 m), warmer seabeds of Kerguelen Plateau, north of frontal activity of Fawn Trough.	19.31	65.53	65.53	605,000
Oates	O	Oceanographically separated from Central Indian-Wilkes subregion with wind & sea ice vectors diverging at western border. Eastern border is adjacent to Ross Sea region.	16.45	40.80	75.71	543,586
Ob & Lena	OL	Shallow, warm seabeds in Polar Frontal Zone, including Ob & Lena banks & seamounts east.	0	0.39	0.39	1,078,842
Pacific Basin	PB	Very deep rugose ocean floor & abyssal plains of South Pacific Ocean Basin warmer than other deep ocean basin regions of Southern Ocean.	2.85	2.85	2.85	3,988,040
Pacific-Antarctic Ridge	PAR	Pacific-Antarctic Ridge region with large extents of shallower environments of depths <2000 m.	11.58	17.89	17.89	3,029,157
Ross Sea	RS	Very cold seabed & high sea ice duration of Ross Sea. 22 endemic molluscs (11.5% of documented species). 16% of documented gastropods endemic.	64.60	77.82	77.82	828,471
South Atlantic	SA	Shallower environments of Mid Atlantic Ridge & associated seamounts	0	0	0	1,908,771
South Georgia	SG	Productive, shallow environments in Polar Frontal Zone including island ecosystems of South Georgia & seamounts of North Scotia Ridge. 65 endemic molluscs (32.7% of documented species). 15% of documented Cheilostomata endemic. 13% of documented bivalves endemic. 36% of documented gastropods endemic.	1.98	34.05	34.05	1,727,252
South Orkney Islands	SOI	Island ecosystems of South Orkney Islands & seamounts & plateaus of South Scotia Arc, many which underlie Southern Antarctic Circumpolar Current Frontal Zone. 22 endemic molluscs	13.17	32.75	43.19	863,550
South Sandwich Islands	SSI	Highly productive island ecosystems of South Sandwich Islands & deeper waters of South Sandwich Trench.	35.23	99.95	99.95	340,884
Weddell Shelf	WS	Very cold seabed & high sea ice duration of Weddell shelf, usually rather deep, ~500–1000 m. 55 endemic molluscs (19.7% of documented species). 26% of documented gastropods endemic.	0	0	83.72	1,257,192

### Pelagic regionalization

Pelagic cluster data representing 20 different categories [46] was downloaded from [70]. Following the methods of two previous pelagic Southern Ocean regionalizations [24, 71], the pelagic regionalization data we used was based on a non-hierarchical clustering algorithm to reduce the number of grid cells, followed by further refinement using a hierarchical clustering algorithm [46]. During the latter, clusters comprised of only one datum were merged into parent clusters (which occurred in five instances in cluster groups 2, 3, 8 and 13). The regionalization used summer climatological sea surface temperature [63], depth [72] and the proportion of time covered by sea ice as input variables [64]. These data were originally calculated south of 40°S, but were bound to the CCAMLR Convention area for this analysis (Table 3). For further details on methods underpinning the generation of the pelagic regionalization data used in this analysis see [46].

**Table 3:** Pelagic clusters of the Southern Ocean, including description (from [46]) and percentages of the pelagic clusters included in: No-take zones of existing MPAs; existing MPAs; and existing and proposed MPAs combined. SST refers to sea surface temperature (note that cluster 18 is not within the CCAMLR Area).

Pelagic cluster	Description	% in no-take	% in MPAs	% in MPAs + proposals	Size of cluster (km^2^)
1	Polynya margins on continental shelf, South Orkneys plateau & areas off Adelaide & Biscoe Island in west Antarctic Peninsula. Moderately shallow (to ~1000 m) with ice cover ~20–50% & SST (<2°C).	6.33	7.23	50.65	283,533
2	Polynyas on continental shelf & areas off Danco Coast of Peninsula & South Orkney Islands & part of Banzare Bank. Low ice cover (~0–20%) & cold SST (<2°C).	1.41	2.43	69.83	165,969
3	Shallow shelf areas with ~25–60% ice cover. Restricted distribution, generally limited to East Antarctica.	4.24	4.55	49.34	30,466
4	Shallow areas with high ice cover (~75–95%). Patchy distribution scattered around continental shelf	17.68	27.30	51.19	37,678
5	Shelf areas with almost perennial ice cover (~75–100%).	5.90	13.62	69.28	1,010,363
6	Similar to 7, but shallower & with lower ice cover. Widely but sparsely distributed around continental shelf	12.83	13.31	51.73	156,512
7	Moderate depths (~200–1000 m) & ice cover (~50–75%). Many areas correspond to regions around polynyas. Also southern Scotia Arc areas.	28.06	32.55	51.02	1,030,815
8	Sea ice zone. Clusters 8–11 form an approximately latitudinal, deep water continuum of increasing ice cover and decreasing SST. Northernmost limit (of cluster 10) is generally just south of mean maximum winter sea ice extent.	23.48	30.98	54.21	1,676,534
9	Sea ice zone. Clusters 8–11 form an approximately latitudinal, deep water continuum of increasing ice cover & decreasing SST. Northernmost limit (of cluster 10) is generally just south of mean maximum winter sea ice extent.	6.91	8.03	26.36	5,178,744
10	Sea ice zone. Clusters 8–11 form an approximately latitudinal, deep water continuum of increasing ice cover & decreasing SST. Northernmost limit (of cluster 10) is generally just south of mean maximum winter sea ice extent.	1.73	5.66	13.04	3,440,399
11	Sea ice zone. Clusters 8–11 form an approximately latitudinal, deep water continuum of increasing ice cover & decreasing SST. Northernmost limit (of cluster 10) is generally just south of mean maximum winter sea ice extent.	2.13	4.03	15.39	3,575,726
12	Moderate depth (~1000–2500 m) & sea ice cover (~40%). Restricted to parts of southern Scotia Arc & isolated pockets north of Balleny Islands & off West Ice Shelf.	11.01	41.67	45.24	47,493
13	Shallow (~200–1000 m) parts of northern Kerguelen, Crozet & South Georgia plateau areas, Conrad Rise.	34.30	79.14	79.16	357,564
14	Deeper (~500–2000 m) parts of same plateaus, also Bouvetøya & northern tip of southern Kerguelen plateau.	3.19	25.93	34.61	322,906
15	Deep oceanic waters, encompassing approximately southern Antarctic Circumpolar Current front & Polar Front.	1.19	6.89	8.07	12,780,390
16	Deep oceanic waters, bounded approximately on north by Sub-Antarctic Front.	1.55	26.99	26.99	3,397,347
17	Temperate waters	2.74	6.98	6.98	255,758
18	Temperate waters	0	0.00	0.00	0
19	Outer areas of South American, New Zealand & Tasmanian shelves & scattered temperate banks.	0	93.28	93.28	657
20	Broad distribution around South American, New Zealand, Tasmanian & Crozet shelves. Shallow, ice-free & with warm SST (~10–20°C).	35.29	99.78	99.78	19,872

### Total proportion of protected area

Using ArcGIS (version 10.5) [73], we calculated the sizes, including of designated no-take areas, in kilometres^2^, of all existing and proposed MPAs. Shapefiles for the CCAMLR Area boundaries, CCAMLR MPAs (South Orkney Islands Southern Shelf and Ross Sea region MPAs) and CCAMLR MPA planning domains were downloaded from [48]. Shapefiles for all nationally governed sub-Antarctic MPAs falling within in our study area (Kerguelen Island, Crozet Island, Prince Edward Islands, Heard Island and McDonald Islands, South Georgia and South Sandwich Islands) were downloaded from [74–77]. The East Antarctic MPA proposal boundaries were drawn based on [49], Weddell Sea MPA boundaries were based on [50], and Domain 1 (Antarctic Peninsula) proposed MPA boundaries were based on [51] (the latter two with permission). All CCAMLR Area and MPA shapefiles were imported into ArcGIS and projected into ESRI:102020 projection, South Pole Lambert Azimuthal Equal Area [73]. Sub-Antarctic MPAs with boundaries extending outside the CCAMLR Area (Kerguelen, Crozet and Prince Edward Islands) were constrained to the CCAMLR Area. We then calculated the total area encompassed by each existing and proposed MPA in ArcGIS. The total proportion of protected area is given by:

$$\sum_{i=100*}^{n}A_{pi}/A$$ (1)

where *A*_*pi*_ = area of each MPA, indexed by *i*, located within the CCAMLR Convention area; *n* is the number of MPAs; and *A* is the total CCAMLR Area. This metric was calculated for each of the nine CCAMLR MPA planning domains as well as for the entire CCAMLR Area. To report the proportion as a percentage, we multiplied the total proportion by 100.

This metric was also calculated for the no-take areas in the CCAMLR Area. To calculate the total proportion of no-take area, *A*_*pi*_ = the no-take area of each MPA, indexed by *i*, located within the target CCAMLR Convention area. As with the total MPA area, we calculated the no-take metric for each CCAMLR MPA planning domain as well as the entire CCAMLR Convention area.

### Fraction of ecoregion protected

We calculated the area and proportion of each benthic and pelagic ecoregion that falls within the boundaries of existing and proposed MPAs, including ecoregions encompassed by no-take zones. Benthic ecoregion and pelagic cluster files (see above) were downloaded and projected into the ESRI:102020 projection, South Pole Lambert Azimuthal Equal Area [73]. The pelagic regionalization was originally projected out to 40°S, thus we constrained the data to the CCAMLR Area. Pelagic cluster 18 fell outside the area of analysis as it only occurs north of the CCAMLR Area. The benthic and pelagic ecoregion data files were intersected with the MPA shapefiles. We then calculated ecoregion areas included in each existing and proposed MPA, including areas encompassed by no-take zones. Mean fraction of each ecoregion protected =

∑1n(∑1mjApij/Aj)n (2)

Where *m*_*j*_ is the number of MPAs in ecoregion or pelagic cluster *j* and *A*_*pij*_ is the area of each MPA, *i*, overlapping areas of ecoregion or pelagic cluster *j*. *A*_*j*_ is the total area of ecoregion or pelagic cluster *y*. We calculated this for both existing and existing + proposed MPAs. This metric was also calculated for the no-take areas in each existing MPA. Note: *n* = 1–23 for the benthic analysis and 1–20 for the pelagic analysis (representing 23 benthic ecoregions and 20 pelagic clusters).

We also calculated the number of benthic ecoregions and pelagic clusters that have at least 10% of their total area protected (per Aichi Target 11 [15]) and at least 30% of their total area protected (per IUCN guidelines [16]). This = the number of times that $\left(\sum_{1}^{m_{j}}A_{pij}/A_{j}\right)$ is ≥ 0.1 and ≥ 0.3, respectively. We calculated this percentage for existing MPAs, existing + proposed MPAs, and no-take zones within existing MPAs.

### Protection equality

Finally, we calculated the protection equality of the existing and proposed MPA system using parallel methods to [22]. These metrics were developed by [78] and are measures of how equitably the different benthic ecoregions and pelagic clusters are represented in the MPA system (i.e., a Gini coefficient). We used the “ProtectEqual” package in R (version 3.5.1) [79], developed by [80], to calculate protection equality values based on the proportion of each ecoregion and pelagic cluster protected. Protection equality values can range from 0–1 with high numbers indicating a higher protection equality.

## Results

### Total proportion of protected area

Seven MPAs currently exist in the Southern Ocean resulting in 11.98% of the CCAMLR Area falling under general protection and 4.61% falling under strict no-take protection (Table 1; Fig 1). Of the 11.98% area protected, nationally managed MPAs account for more than half of this (7.21%) and CCAMLR-governed MPAs account for the latter (4.6%). MPAs implemented in the CCAMLR Area which fall under national jurisdiction are: the Heard Island and McDonald Islands (HIMI) marine reserve (~71,000 km^2^; adopted in 2002 and expanded in 2014; governed by Australia), the South Georgia and South Sandwich Islands MPA (~1.24 million km^2^; adopted in 2012 and expanded in 2019; governed by the United Kingdom), the Prince Edward Islands MPA (~180,000 km^2^; adopted in 2013; governed by South Africa), and the Crozet and Kerguelen Islands MPAs (~1.14 million km^2^; adopted in 2017; governed by France). Note that the northern boundaries of the Prince Edward Islands, Kerguelen and Crozet MPAs extend beyond the CCAMLR Convention Area boundary. CCAMLR has also collectively adopted two MPAs: the South Orkney Islands Southern Shelf MPA (~94,000 km^2^; adopted in 2009) and the Ross Sea region MPA (~1.55 million km^2^; adopted in 2016). Three large MPA proposals also remain under negotiation at CCAMLR in the East Antarctic (proposed at ~1 million km^2^), the Weddell Sea (~2 million km^2^) and in Domain 1 (~466,000 km^2^) (Fig 1).

Of the nine planning domains established by CCAMLR (Fig 1), existing MPAs cover parts of Domain 1 (South Orkney Islands Southern Shelf MPA), Domain 2 (South Georgia and South Sandwich Islands MPA), Domain 5 (Prince Edward Islands and Crozet MPAs), Domain 6 (Kerguelen and HIMI MPAs), and Domain 8 (Ross Sea region MPA). Proposed MPAs would further cover Domain 3 and 4 (Weddell Sea MPA), Domain 7 (East Antarctic MPA), and additional areas in Domain 1 (Domain 1 MPA). Domain 9 remains un-represented (Fig 1).

### Fraction of ecoregion protected

**Fig 2:**
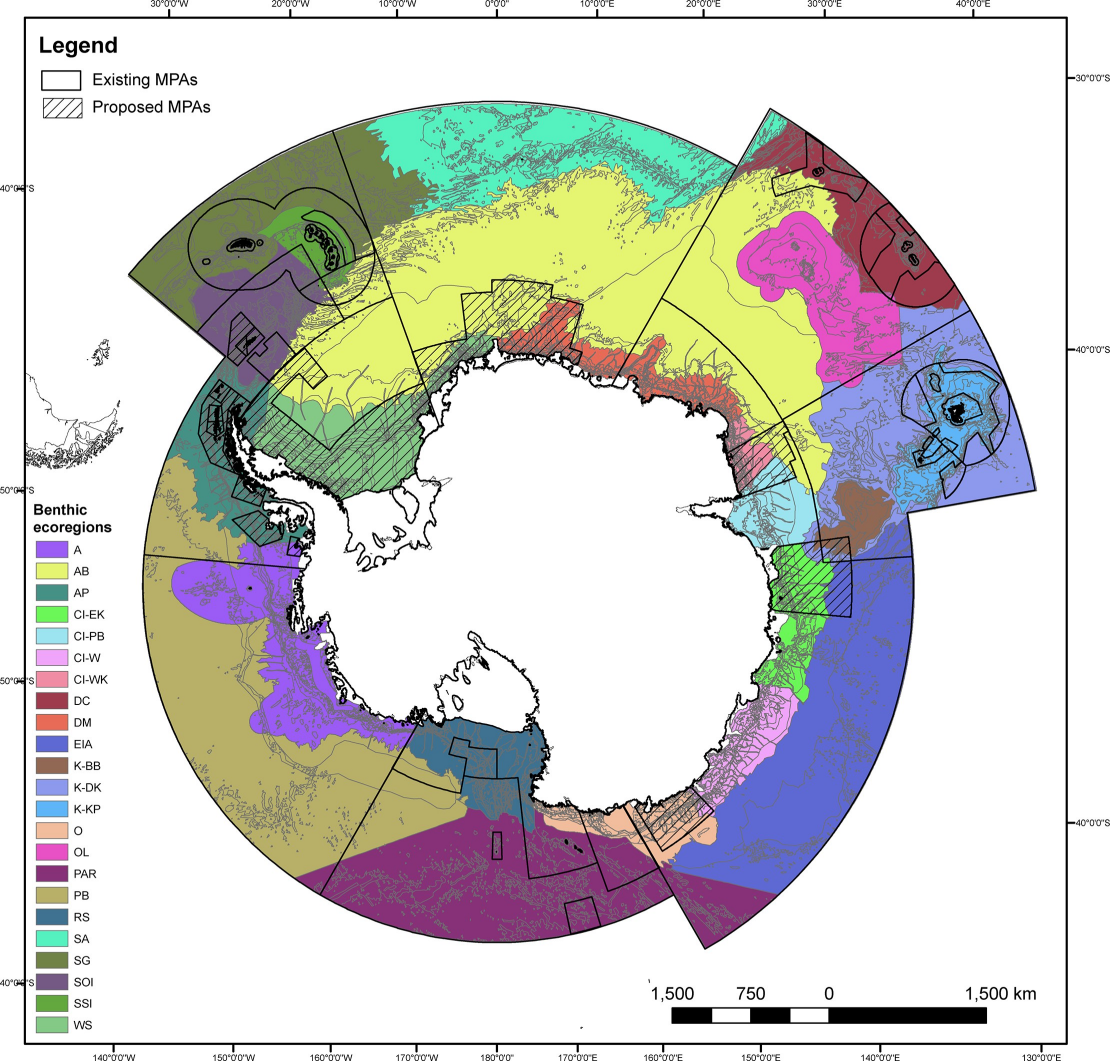
Benthic ecoregions of the CCAMLR Area and marine protected areas. Benthic ecoregions [45], with existing and proposed MPAs overlain (in black outline and hashed line, respectively; no-take regions outlined in existing MPAs; see Fig 1 for delineation). Benthic ecoregion abbreviations defined in Table 2.

In the CCAMLR Area, 23 benthic ecoregions have been identified [45]. Of these, 12 benthic ecoregions are at least partially protected in no-take zones of existing Southern Ocean MPAs (0.21–64.0%; median = 7.58, mean = 14.33; Table 2). However, only six of these benthic ecoregions have 10% or more no-take protection; only two benthic ecoregions have 30% no-take protection (Table 4). Across all zones of existing Southern Ocean MPAs, 13 benthic ecoregions are at least partially represented in existing MPAs (0.21–99.95%; median = 32.75; mean = 34.22; Fig 2; Table 2). Nine of these benthic ecoregions are at least 10% represented in existing MPAs; seven benthic ecoregions are at least 30% represented in the existing MPAs (Table 4). Ten benthic ecoregions are not represented in the current Southern Ocean MPA network (Fig 2; Table 2).

**Table 4:** Number of benthic ecoregions and pelagic clusters that have ≥ 10% and ≥ 30% represented (out of 23 benthic ecoregions and 19 pelagic clusters considered in this analysis). See Table 2 and Table 4 for names and descriptions of benthic ecoregions and pelagic clusters.

10% THRESHOLD								
	No-take zone (in existing)		Existing MPA		Existing + Proposed		Not at threshold (in existing + proposed)	
*Total*	6		9		17		6	
*Benthic Ecoregions*	K-KP	RS	DC	RS	AB	K-KP	A	OL
	O	SOI	K-DK	SG	AP	O	CI-W	PB
	PAR	SSI	K-KP	SOI	CI-EK	PAR	EIA	SA
			O	SSI	CI-PB	RS		
			PAR		CI-WK	SG		
				DC	SOI			
				DM	SSI			
				K-BB	WS			
				K-DK				
*Total*	7		11		17		2	
*Pelagic clusters*	4	12	4	13	1	10	15	
	6	13	5	14	2	11	17	
	7	20	6	16	3	12		
	8		7	19	4	13		
			8	20	5	14		
			12		6	16		
					7	19		
					8	20		
					9			
**30% THRESHOLD**								
*Total*	2		7		12		11	
*Benthic ecoregions*	RS		DC	SG	AP	O	A	K-DK
	SSI		K-KP	SSI	CI-EK	RS	AB	OL
			O	SOI	CI-WK	SG	CI-PB	PB
			RS		DC	SSI	CI-W	PAR
					DM	SOI	EIA	SA
					K-KP	WS	K-BB	
*Total*	2		6		13		6	
*Pelagic Clusters*	13		7	13	1	8	9	15
	20		8	19	2	12	10	16
			12	20	3	13	11	17
					4	14		
					5	19		
					6	20		
					7			

Designation of the proposed MPAs in the East Antarctic, Weddell Sea and Domain 1 (Antarctic Peninsula) currently being negotiated by CCAMLR would provide representation of an additional nine benthic ecoregions. This would increase the total to 22 of the 23 benthic ecoregions (all except for the South Atlantic ecoregion), at least partially, within protected areas (1.15–99.95%; median = 34.40; mean = 37.44; Fig 2; Table 2). Inclusion of these additional proposed MPAs in the Southern Ocean MPA network would result in 17 benthic ecoregions being at least 10% protected; 12 of these benthic ecoregions would achieve being at least 30% protected (Table 4).

**Fig 3:**
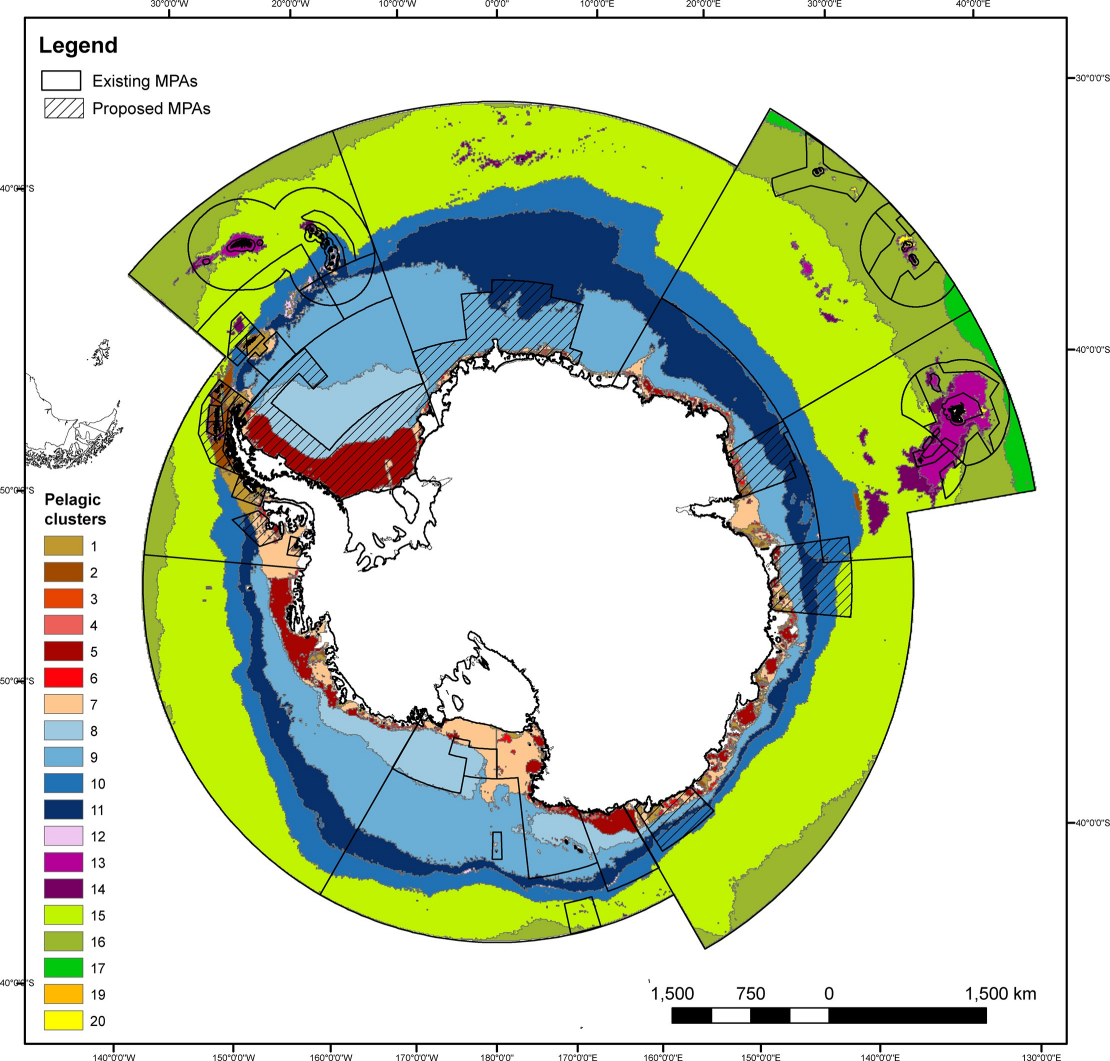
Pelagic clusters of the CCAMLR Area and marine protected areas. Pelagic clusters [46], with existing and proposed MPAs overlain (in black outline and hashed line, respectively; no-take regions outlined in existing MPAs; see Fig 1 for delineation). Pelagic cluster numbers defined in Table 3 (note that cluster 18 only occurred outside the CCAMLR Area, thus outside the scope of this analysis).

In the Southern Ocean, 20 pelagic clusters have been identified [46], however only 19 of these fall within the CCAMLR Area (cluster 18 only occurs outside the CCAMLR Area). Of these, 18 pelagic clusters are at least partially protected in no-take regions of existing Southern Ocean MPAs (1.19–35.29%; median = 6.11; mean = 11.11; Table 3). However, only seven of these pelagic clusters are at least 10% protected in no-take zones; only two are at least 30% protected in no-take zones (Table 4). In all zones of established Southern Ocean MPAs, all of the 19 pelagic clusters are at least partially represented in the seven existing Southern Ocean MPAs (2.43–99.78%; median = 13.62; mean = 27.91; Fig 3; Table 3). Eleven of these pelagic clusters are at least 10% represented in existing MPAs; six are at least 30% represented in existing MPAs (Table 4).

Proposed MPAs in the East Antarctic, Weddell Sea and Domain 1 (Antarctic Peninsula) currently being negotiated by CCAMLR would increase representation of almost all pelagic cluster types (6.98–99.78%; median = 50.65; mean = 47.17; Fig 3; Table 3). Including these additional proposed MPAs in the Southern Ocean MPA network would result in 17 of the pelagic clusters being at least 10% protected; and 13 of the clusters being at least 30% protected (Table 4).

### Protection equality

The protection equality of the no-take zones of existing MPAs were 0.18 and 0.41 for benthic ecoregions and pelagic clusters, respectively (Table 5). For all zones of existing MPAs, the protection equality values increased to 0.26 and 0.44 for benthic and pelagic regions, respectively. Including the existing and the proposed MPAs increased the protection equality values to 0.52 and 0.67 for benthic and pelagic regions, respectively (Table 5).

**Table 5:** Protection equality and integrated protection values of the existing and proposed MPA system, including no-take zones.

PROTECTION EQUALITY			
	No-take	Existing MPA	Existing + Proposed
Benthic ecoregion	0.18	0.26	0.52
Pelagic cluster	0.41	0.44	0.67

## Discussion

CCAMLR has successfully adopted two MPAs in the Southern Ocean, with the Ross Sea being the world’s largest international MPA at ~1.55 million km^2^. CCAMLR jurisdiction MPAs encompass 4.6% of the CCAMLR Area, mostly comprised by the large Ross Sea region MPA. Nationally implemented MPAs encompass 7.21% of the CCAMLR Area. Collectively almost 12% of the Southern Ocean is encompassed in MPAs, thus the region meets the 10% area targets of the Convention on Biological Diversity [15] and the United Nations Sustainable Development Goals [17], and in surpassing the proportion ice-free areas protected on the Antarctic continent [22]. No other high seas management body has achieved this level of protection. It exceeds the global average of 7.91% [36]. Many national waters have not reached the 10% target (e.g., Norway at 0.83%), however, others have far surpassed it (e.g., United States, France, and Australia all have greater than 40% of their national waters protected) [36]. Indeed, among the 66 large marine ecosystems in the world, the Antarctic has the 2^nd^ largest area encompassed by MPAs and the Ross Sea MPA is considered to contain a high level of ecological representativeness for Antarctic biodiversity [37].

Despite having more than 10% of the Southern Ocean protected, only 4.61% is encompassed in no-take areas, largely comprised of the South Orkney Islands Southern Shelf MPA, HIMI marine reserve, and a large proportion (~70%) of the Ross Sea region MPA. Multiple studies point to the importance of MPAs having no-take areas to be effective at conserving biodiversity, including fish populations [4, 5, 81–83]. Furthermore, the Ross Sea region MPA has a limited 35-year duration, meaning that this proportion might not receive protection after this time if the MPA is not renewed. Moreover, while some targets call for 10% protection, many studies suggest that less than 30% is insufficient to protect biodiversity, conserve ecosystem services–including sustaining commercial fisheries–and to achieve socioeconomic priorities set by these targets [9, 84, 85]. Others have argued that protection targets closer to 50% protection are required to curb biodiversity loss [18, 86, 87].

Beyond percentage targets, current protected areas do not provide a representative sample of the Southern Ocean’s biodiversity. Global targets call for protected areas to be ecologically representative [14–16], meaning that protection should encompass the full range of biodiversity present in a region [88]. Overall, current MPA distribution is largely biased towards sub-Antarctic regions and the Ross Sea. Thus, within currently established no-take areas in the Southern Ocean, only two benthic ecoregions have 30% protection. The Ross Sea ecoregion meets this threshold, due to the large-scale MPA in that region and the South Sandwich Islands ecoregions also has this level of protection due to recent (2019) expansions in no-take areas [55]. For pelagic clusters, only 13 (shallow parts of sub-Antarctic plateaus near Kerguelen and South Georgia) and 20 (Crozet shelves) have 30% protected in sub-Antarctic MPAs. At the 10% threshold, still only six benthic ecoregions and seven pelagic clusters are protected in no-take areas. Factoring in all existing MPAs, including no-take and multi-use zones, the Southern Ocean MPA network is still not representative of all benthic ecoregions and pelagic clusters, thus it is not representative of Southern Ocean biodiversity. This is in line with global MPA trends where, while there has been an overall increase in representation, overall 61% of the benthic ecoregions in national waters remain unprotected [38] and globally, most large-marine ecosystems do not have greater than 10% representation [37].

The adoption of additional proposed MPAs in the Weddell Sea, East Antarctic and Antarctic Peninsula would increase representation in the Southern Ocean MPA network. All of these regions encompass parts of CCAMLR’s MPA planning domains (Fig 1) and original priority areas [89]. With the addition of these proposed MPAs, roughly three-quarters of the benthic ecoregions and almost all pelagic clusters would be 10% represented. However, as noted above, to conserve biodiversity these additional MPAs should have no-take zones and further should have long duration (e.g., [81]). Even with the addition of pending MPA proposals, some areas remaining poorly represented. These include the benthic ecoregions of the South Atlantic (mostly in northern Domain 4), Amundsen (mostly in Domain 9), Central Indian-Wilkes subregion (in Domain 7), East Indian Abyssal (mostly in Domain 7), Ob and Lena (mostly in Domain 5), and Pacific Basin (largely in Domain 9). Additional MPAs (to those existing or currently being proposed) would allow for complete representation.

The Southern Ocean MPAs also do not have equitable protection in terms of proportionality protected across benthic ecoregions. For existing MPAs, the benthic ecoregions fall within the lowest quartile and the pelagic clusters are in the second lowest quartile for equality protection [78],. However these numbers are comparable to protection equality values for national MPAs globally [38]. While these values are much higher for the network of MPAs achieved by currently existing and proposed MPAs, protection equality still measures at less than 50% for benthic ecoregions and 60% for pelagic clusters which puts them in the second highest quartile [78]. While not completely equitable, these values are much higher than the values for MPAs globally inside national waters [38].

This assessment of representativeness was undertaken on the basis of large-scale benthic and pelagic regionalizations. The large-scale regions provide a helpful broad measure of progress but do not go far enough to plan for capturing biodiversity patterns, internal heterogeneity, genetic diversity and cryptic species [45]. Although biological and ecosystem-level data are more difficult to work with, and typically do not have consistent circumpolar coverage, consideration of such data might also provide a more nuanced assessment of the strengths and gaps in current and proposed MPAs (e.g., [90–92]). Furthermore, at smaller scales, MPAs may be designed to protect vulnerable or critical habitats that are missed in broad-scale regionalizations. Ensuring protection of all ecoregions and replicated protection of particular ecoregions across different ocean basins is one possible means of addressing this.

The urgency of the threats to the Southern Ocean and the need for protection is critical now more than ever before. The Southern Ocean supports international commercial fisheries for Patagonian and Antarctic toothfish (*Dissostichus eleginoides* and *D*. *mawsoni;* sold as Chilean sea bass) and Antarctic krill (*Euphausia superba*) [44]. Pressure on these fisheries has increased in recent years [93] and is likely to continue, and at the same time climate change pressures on Southern Ocean ecosystems are also increasing [94–98]. The cumulative impacts of fishing and climate change are likely to have greater effect than either impact alone [99–101]. Increasing numbers of studies show that MPAs, especially no-take marine reserves, can be a proactive and precautionary tool to enhance resilience to environmental change, including climate change and warming [10–12, 102, 103]. Importantly, the MPAs need to be well designed, with representation being one of many elements. Key biodiversity areas, vulnerable and rare species should be considered in MPA design, as well as connectivity (e.g., [6, 104]). Further, the MPAs need to be well managed and enforced [105, 106], a significant challenge for large-scale MPAs in a place as large and remote as the Antarctic [107].

Nonetheless, protected areas alone will not suffice to conserve Antarctic marine biodiversity [108]. CCAMLR may need to enact other precautionary management measures targeted at reducing or even eliminating fish catch in some areas [109]. Given the international nature of climate change and threats to Antarctic biodiversity, successful deployment of such measures by CCAMLR will require collaboration with other appropriate international organizations and initiatives, including those of the United Nations [110–113]. Integration across these management bodies will broaden CCAMLR’s toolbox [114] for taking action on conserving the globally significant biodiversity and living resources of the Southern Ocean [115–118].
